# VOC Contamination in Hospital, from Stationary Sampling of a Large Panel of Compounds, in View of Healthcare Workers and Patients Exposure Assessment

**DOI:** 10.1371/journal.pone.0055535

**Published:** 2013-02-05

**Authors:** Vincent Bessonneau, Luc Mosqueron, Adèle Berrubé, Gaël Mukensturm, Sylvie Buffet-Bataillon, Jean-Pierre Gangneux, Olivier Thomas

**Affiliations:** 1 Environmental and Health Research Laboratory, Advanced School of Public Health, Rennes, France; 2 U 1085 Institute of Research in Environmental and Occupational Health, University of Rennes 1, Rennes, France; 3 Veolia Environment Research & Innovation, Rueil-Malmaison, France; 4 Operational Staff for Hospital Hygiene, Centre Hospitalier Universitaire de Rennes, Rennes, France; 5 Parasitology-Mycology Laboratory, Centre Hospitalier Universitaire de Rennes, Rennes, France; Chemistry, Sweden

## Abstract

**Background:**

We aimed to assess, for the first time, the nature of the indoor air contamination of hospitals.

**Methods and Findings:**

More than 40 volatile organic compounds (VOCs) including aliphatic, aromatic and halogenated hydrocarbons, aldehydes, alcohols, ketones, ethers and terpenes were measured in a teaching hospital in France, from sampling in six sampling sites – reception hall, patient room, nursing care, post-anesthesia care unit, parasitology-mycology laboratory and flexible endoscope disinfection unit – in the morning and in the afternoon, during three consecutive days. Our results showed that the main compounds found in indoor air were alcohols (arithmetic means ± SD: 928±958 µg/m^3^ and 47.9±52.2 µg/m^3^ for ethanol and isopropanol, respectively), ethers (75.6±157 µg/m^3^ for ether) and ketones (22.6±20.6 µg/m^3^ for acetone). Concentrations levels of aromatic and halogenated hydrocarbons, ketones, aldehydes and limonene were widely variable between sampling sites, due to building age and type of products used according to health activities conducted in each site. A high temporal variability was observed in concentrations of alcohols, probably due to the intensive use of alcohol-based hand rubs in all sites. Qualitative analysis of air samples led to the identification of other compounds, including siloxanes (hexamethyldisiloxane, octamethyltrisiloxane, decamethylcyclopentasiloxane), anesthetic gases (sevoflurane, desflurane), aliphatic hydrocarbons (butane), esters (ethylacetate), terpenes (camphor, α-bisabolol), aldehydes (benzaldehyde) and organic acids (benzoic acid) depending on sites.

**Conclusion:**

For all compounds, concentrations measured were lower than concentrations known to be harmful in humans. However, results showed that indoor air of sampling locations contains a complex mixture of VOCs. Further multicenter studies are required to compare these results. A full understanding of the exposure of healthcare workers and patients to complex mixtures of chemical compounds can then be related to potential health outcomes.

## Introduction

Besides microbial contamination with hundreds of research papers in relation to hospital-acquired infections, the chemical contamination of indoor air in hospitals is rarely studied. Taking into account the specificity of hospital activities, healthcare workers (HCWs) and patients may be exposed to a wide range of chemical compounds emitted from various products such as disinfectants and sterilitants (ethylene oxide, glutaraldehyde, formaldehyde, alcohols…), anesthetic gases, laboratory or pharmaceutical products [1]. Some studies have shown that HCWs reported more indoor air-related symptoms than people working in office buildings [2]. In addition, a lower prevalence of indoor air-related symptoms were reported in hospitals, where workers perceived a good indoor air quality (IAQ) [3], [4]. Other studies have reported that exposure of HCWs to disinfectants and sterilitants (glutaraldehyde, hydrogen peroxide, alcohols) could induce allergic reactions such as conjunctivitis, rhinitis or contact dermatitis [5]. Exposure to glutaraldehyde could also be associated to occupational asthma [6]. Most of studies have assessed the exposure of HCWs only to a few compounds such as anesthetic gases (operating rooms), formaldehyde (pathology laboratories), or glutaratldehyde and ethylene oxide (disinfection units). Dascalaki et al. [7] have reported an average concentration of 8,8862 µg/m^3^ for total volatile organic compounds (TVOC) in operating rooms with a contamination dominated by anesthetic gases (isoflurane and sevoflurane). Others compounds identified were aldehydes (formaldehyde, glutaraldehyde), aromatic hydrocarbons (benzene, toluene, xylene, ethylbenzene and dimethylbenzene), alcohols and oxides. A few studies have assessed the indoor air quality in different hospital areas. Ghasemkhani et al. [8] have reported that the concentrations of formaldehyde were higher in pathology laboratories than those measured in surgery rooms and endoscopy wards. High levels of ethylene oxide were observed in disinfection and sterilization units for certain situations [9]. In a newly constructed hospital, TVOC concentrations were higher than 400 µg/m^3^, in nearly half of the patients rooms studied [10]. Some studies have reported the presence of other compounds including acetone, acetaldehyde, 2-butanone [11], [12], 2-phenoxyethanol, butoxyethanol, hexanal and nonanal [13].

Exposure to VOCs is of particular concern due to their potential adverse health effect on humans. Some of them have received regulatory attention. Among aromatic hydrocarbons, benzene is classified as carcinogenic to humans by IARC (group 1), while there is limited evidence of carcinogenicity in humans for ethylbenzene and styrene (classified in the group 2B by IARC). For xylenes (o,m,p), toluene and phenol, the evidence of carcinogenicity in humans is inadequate (classified in the group 3 by IARC). The classification of trichloroethylene and 1,4-dichlorobenzene as probably carcinogenic for humans by IARC is based on sufficient evidence of a positive association between exposure to these compounds and the development of cancers, in experimental animals. Exposure to chloroform has been associated to the development of liver and kidney cancers, in experimental animals that lead the IARC to classify this substance as possibly carcinogenic to humans. Among aldehydes, adverse health effects of formaldehyde are well known. Formaldehyde has been classified in 2004 from possibly carcinogenic to humans to carcinogenic to humans, based on epidemiological evidences of the development of nasopharyngeal cancer. Acetaldehyde is suspected to increase the risk of bronchial and oral cavity tumors, but there is inadequate evidence in humans. It has been classified as possibly carciogenic to humans by IARC. For acrolein, there is limited evidence for its carcinogenicity in experimental animals. For other aldehydes, no data are available.

Besides the previous chemicals, the use of alcohol-based hand rubs (ABHRs) is highly recommended for hand hygiene to reduce hospital-acquired infections. Most commercially available ABHRs contain 70% by weight of ethanol and isopropanol [14]. During hygienic hand disinfection, users would be exposed, for short periods, to a sudden change in ethanol concentrations from 0 to 14.3 mg/L [15]. Semi-volatile organic compounds (SVOCs), such as phatlates, could also be released from PVC-based medical devices. Among phtalates, DEHP and DBP are of great concern since they are suspected to be endocrine disruptors [16], or induce respiratory and allergic disorders [17].

In hospitals, as in other buildings, indoor air quality could be affected by the emission of some VOCs and SVOCs from building and decoration materials [18], outdoor air (e.g. vehicle emission) [19], and micro-organims such as bacteria and fungi [20].

The wide diversity of health activities conducted in a hospital could induce a high heterogeneity of the indoor air contamination and there is a lack of knowledge about chemical contamination, both in terms of nature and concentration levels.

This study aims at proposing a methodology for the assessment of the nature of the VOCs contamination of hospital indoor air in different locations. 42 VOCs, including aldehydes, alcohols, ketones, aromatic, aliphatic and halogenated hydrocarbons, ethers and terpenes were quantified. Qualitative analysis of samples was also carried out to detect other compounds.

## Materials and Methods

### Study design

The study was conducted during three consecutive days in March 2012 at the teaching hospital of Ponchaillou in Rennes, France. This hospital is a 1,952-bed tertiary hospital and surgical facility. Air samples were collected in six sites of the hospital – the reception hall, a patient room, a nursing care, the parasitology-mycology laboratory, a post-anesthesia care unit (PACU), and the flexible endoscope disinfection unit – in order to estimate the spatial variability in VOCs concentrations in indoor air. Since no outdoor air samples were collected, the reception hall was selected as a control site where the indoor air contamination is mainly due to sources not related to healthcare activities (e.g. building materials or outdoor air). To assess the daily and weekly variations in the chemical contamination, six sampling sessions were conducted: morning and afternoon during three days (Sunday, Monday, and Tuesday). For each sampling session, air samples were collected during 3 h at the breathing zone (i.e. 1.5 m).

### List of products used in the hospital

In order to optimize the study design and the selection of compounds to include in the measurement campaign, a preliminary interview was carried out among hospital staff. A list of products used, the volume and their frequency of use, as well as their formulation and the capacity of active substances to volatilize, was set up by interviewing 15 people working in the sampling locations and at the purchasing department [21]. Products were classified into five groups (laboratory products, cleaning/disinfectants products, alcohol-based products, pharmaceutical products/antiseptics, and anesthetic gases).

### Sampling

A high heterogeneity of the indoor air quality is expected in hospitals due to the large diversity of health activities. The primary purpose of our study is to provide concentration levels of VOCs in different sites of one hospital as well as the temporal variability within each site. Air samples were dynamically collected using a low flow pump LFS 113 DC (GE Industry, Sensing, France), because this sampling method is the most suitable for monitoring concentration change over the time [22]. The pumping flow rate was set, before each sampling session, using a Gilian Gilibrator 2 (GE Industry, Sensing, France) and controlled at the end of the session to ensure that flow rate did not undergo changes. Compared to passive sampler such as Radiello®, active sampling method provides similar analytical performance, without requiring the determination of VOCs uptake rates [23]. As very low concentrations were expected, VOCs were pre-concentrated on solid sorbents. Enrichment was carried out using tubes containing three different carbon-based sorbents, arranged from weak to strong sorption strength, because target VOCs ranged from low volatility to high volatility. Multi-sorbent tubes show better adsorption performance than tubes containing one absorbent for very volatile compounds [24]. In addition, this kind of tubes facilitates the adsorption of VOCs over a wide volatility range [22], [25]. Two different sorbent systems were used, because VOCs analyses were split between two laboratories, which validated their analytical methods using a specific sorbent system. Aromatic and halogenated hydrocarbons, alcohols, and ketones sampling was carried out with a multisorbent tube packed with Carbopack C, Carbopack B and Carboxen 1000 (pumping flow rate of 30 mL/min) whereas aliphatic hydrocarbons, ethers and terpenes sampling was done by multisorbent tube packed with Tenax TA, Carbograph 1 TD and Carboxen 1000 (pumping flow rate of 50 mL/min). For aldehydes, air sample was dynamically collected through a first cartridge containing potassium iodide to prevent the interference of ozone (Sep-Pak® Ozone Scrubber, Waters Corp., Milford, MA, USA), then through a second cartridge containing silica gel coated with 2,4-dinitrophenylhydrazine (DNPH) (Sep-Pak® XpoSure Aldehyde Sampler, Waters Corp., Milford, MA, USA), with a pumping flow rate of 1 L/min. All sampling were conducted simultaneously. After sampling, sorbent tubes and cartdridges were immediately capped and stored at 6°C for up to 2 weeks. Before sampling, a thermal conditioning system (TERA environnement, Crolles, France) was used to clean up 18 tubes simultaneously. Multisorbent tubes were conditioned at 400°C for 20 min with a 70 mL/min N_2_ flow rate. After conditioning, tubes were immediately sealed with brass long-term storage caps and stored at 6°C for up to 2 months.

### VOC Analysis

Standards of VOCs, with purity not less than 98%, were obtained from Aldrich (Milwaukee, WI, USA), Merck (Darmstadt, Germany) and Fluka (Buchs, Switzerland). For the determination of aromatic and halogenated hydrocarbons, alcohols, ketones, and one aldehyde (acrolein), the analysis (method 1) was carried out with an automatic thermal desorption unit (ATD Turbomatrix 650, Perkin-Elmer, Boston, MA, USA) coupled with a capillary gas chromatograph (HP 6890, Hewlett-Packard, Pablo Alto, CA, USA) and a mass spectrometer as detector (Agilent 5975C, Agilent technologies, Santa Clara, CA, USA) whereas, for the determination of aliphatic hydrocarbons, ethers and terpenes, the analysis (method 2) was performed with an automatic thermal desorption unit (Unity 1, Markes International Limited, Llantrisant, UK) coupled with a capillary gas chromatograph (HP 6890, Hewlett-Packard, Pablo Alto, CA, USA) and a mass spectrometer as detector (HP 5973, Hewlett-Packard, Pablo Alto, CA, USA). The ATD/GC/MS optimized parameters are presented in Table 1.

**Table 1:** Parameters for ATD/GC/MS analysis of the method 1 and 2.

		Value	
Step	Analytical parameter	Method 1	Method 2
Primary desorption	Purge time	1 min	1 min
	Desorption time	15 min	15 min
	Desorption temperature	360°C	290°C
	Desorption gaz	N_2_	N_2_
	Desorption flow	50 mL/min	20 mL/min
	Temperature of cold trap	−20°C	25°C
Secondary desorption	Desorption time	15 min	3 min
	Temperature of cold trap desorption (heating rate)	300°C (40°/s)	290°C (40°C/s)
	Temperature transfer line	290°C	140°C
GC analysis	Gas carrier	He	He
	Gas flow	0.8 mL/min	2.5 mL/min
	Capillary column	Rxi 624 Sil MS, 30 m×0.25 mm×0.25 µm	RTX 502.2, 30 m×0.32 mm×1.8 µm
	Oven temperature	40°C for 2 min, 10°C/min up to 220°C 220°C for 5 min	40°C for 10 min, 7°C/min up to 145°C 20°C/min up to 250°C 250°C for 5 min

Qualitative analysis of air samples was conducted following the method 2. The mass scanning in electron impact mode was done for the range of 14–500 m/z at a rate of 3.3 scans/s. Mass spectra were compared to the NIST database for compounds identification.

For the determination of other aldehydes, the DNPH cartdridge was eluted with 5 mL of acetonitrile (ACN). The analysis was carried out with a HPLC system (HP 1100, Agilent Technologies, Santa Clara, CA, USA) coupled to a diode array detector. 25 µL of the extract were directly injected into a Supelco Discovery C18 column (250 mm×4.6 mm, particle size 5 µm, Supelco, Bellefonte, PA, USA) protected by a Vydac® 201TP C18 guard column, heated at 25°C with a flow rate of 1 mL/min. LC separation of carbonyls was conducted using a mixture of ACN and water as mobile phase. The gradient program was as follows: constant 60% water and 40% ACN during 0–20 min, then the content of ACN increased to 80% during 20.1–48 min and kept constant until 53 min, and then restored to 40% during 53.1–60 min. The detection of carbonyls was performed with a UV diode array set at 365.4 nm.

### Method validation

The limit of detection (LOD) was defined as the concentration at which the signal-to-noise ratio (S/N) is equal to 3. The limit of quantification (LOQ) was defined as the lowest concentration at the signal-to-noise ratio of ≥10 with a precision <25%. For each method, the precision was determined in five replicates at the LOQ and given as a percentage of the relative standard deviation (RSD). Table 2 summarizes the validation results for ATD/GC/MS methods. For aldehydes, the LOD was 0.12 µg and the LOQ was 0.37 µg with a precision <15%.

**Table 2:** Limit of detection (LOD), limit of quantification (LOQ), linear dynamic range and precision (% RSD) of the analytical methods used.

Compound	LOD (ng)	LOQ (ng)	Linear dynamic range (ng)	RSD (%)
**ATD/GC/MS Method 1**				
*Aromatic hydrocarbons*				
Benzene	1.9	6.25	6.25–1250	19
Ethylbenzene	1.9	6.25	6.25–1250	13
m,p-Xylene	3.8	12.5	12.5–2500	20
o-Xylene	1.9	6.25	6.25–1250	20
Styrene	0.04	0.12	0.2–40	11
Toluene	1.9	6.25	6.25–625	20
1,2,4-Trimethylbenzene	0.02	0.06	0.1–20	13
Naphtalene	0.6	2	2–400	11
Phenol	0.4	1.3	1.5–300	18
*Halogenated hydrocarbons*				
1,1,1-Trichloroetane	0.2	0.8	1–200	5
Trichloroethylene	0.6	2	2–400	11
Chloroform	0.4	1.25	1.25–250	12
1,4-Dichlorobenzene	0.1	0.3	0.5–100	19
*Alcohols*				
Ethanol	0.6	2.1	80–16000	17
Isopropanol	1.5	4.9	10–2000	18
Propanol	1.4	4.7	5–1000	16
2-Ethyl-1-hexanol	0.2	0.7	1–200	16
2-Phenoxyethanol	0.06	0.2	0.2–40	19
*Aldehyde*				
Acrolein	0.09	0.3	0.3–60	10
*Ketones*				
Acetone	0.03	0.09	5–1000	15
2-Butanone	0.02	0.07	5–1000	8
Cyclohexanone	0.05	0.15	0.5–100	20
**ATD/GC/MS Method 2**				
*Aliphatic hydrocarbons*				
n-Hexane	3	10	10–1000	20
Cyclohexane	3	10	10–1000	9
n-Heptane	3	10	10–1000	2
n-Decane	15	50	50–1000	3
n-undecane	15	50	50–1000	5
*Ethers*				
Ether	3	10	10–1000	2
2-Ethoxyethanol	3	10	10–1000	3
2-Butoxyethanol	15	50	50–1000	9
*Halogenated hydrocarbon*				
1-Bromopropane	3	10	10–1000	NA
*Terpene*				
Limonene	15	50	50–1000	13

NA: Not available.

### Quality assurance and quality control

For VOCs analyzed using ATD/GC/MS methods, the quantification was performed in selected ion monitoring mode (SIM) of MS detection. Calibration solutions containing each compound at six concentration levels were prepared in methanol. 1 µL of calibration solutions were introduced onto the multisorbent tubes using an adsorbent tube injector system (ATIS™, Supelco, Bellefonte, PA, USA) in order to obtain a known amount of target compounds in six multisorbent tubes.

For aldehydes, the quantification was conducted by external calibration method. Calibration solutions containing each compound at seven concentration levels were prepared in ACN.

Calibration points, quality controls and laboratory blanks were analyzed with each set of samples to ensure analytical systems stability as well as results integrity.

### Statistical analysis

Basic statistical exploitation on the collected data was performed using GraphPad Prism version 5.01 for Windows (GraphPad Software, San Diego, CA, USA). Concentrations below LOQ were replaced by the value of LOQ divided by the root square of two. For descriptive statistic purpose, the arithmetic means of concentrations were reported with the standard deviation. Differences among concentrations were evaluated by non-parametric Kruskall-Wallis test for the comparison of three or more parameters.

## Results and Discussion

### List of products used in the hospital

Table 3 presents the list of products used in the hospital.

**Table 3:** List and number of different class of products used in the six sampling sites.

Class of Products	PR	NC	PACU	DU	PL	RH
Laboratory products	0	0	0	0	24	0
Cleaning/disinfectant products	6	6	4	9	8	4
Alcohol-based products	4	4	4	1	0	0
Pharmaceutical product/antiseptics	11	4	1	0	1	0
Anesthetic gases	0	0	3	0	0	0
Total	21	14	12	10	33	4

PR: patient room; NC: nursing care; PACU: post-anesthesia care unit; DU: endoscope disinfection unit; PL: parasitology laboratory; RH: reception hall.

Our study listed 58 different products used in the six sampling sites. Laboratory products were the most used products (41%), followed by cleaning and disinfectants (28%), pharmaceutical products/antiseptics (19%), alcohol-based products (7%), and anesthetic gases (5%). The use of products listed was specific to health activities conducted in the different sampling sites. Cleaning/disinfectants and alcohol-based products were used in most of sites, in order to reduce hospital-acquired infections. The parasitology laboratory was the site with the highest number of products used, among which 73% were strictly laboratory products (chemicals and reagents). A high number of products were also used in the hospital room, with 11 pharmaceutical products/antiseptics for patients care. Three different anesthestic gases were used in the PACU.

### Spatial and temporal variability in concentrations of target compounds

Table 4 presents the distribution of indoor air concentrations of target compounds measured in all sites.

**Table 4:** Distribution (mean, standard deviation (SD), minimum, 25^th^ percentile, median, 75^th^ percentile and maximum) of indoor air concentrations of target compounds measured in all sites (*n* = 36).

		Concentration (µg/m^3^)					
Compounds	<LOQ (%)	Mean (SD)	Min	25^th^ p.	50^th^ p.	75^th^ p.	Max
**Aromatic hydrocarbons**							
Benzene	71	1.6 (1.5)	0.5	0.6	0.8	2.3	5.1
Ethylbenzene	54	1.8 (1.8)	0.1	0.7	0.8	2.9	6.6
*m,p*-Xylene	51	3.6 (3.1)	1.0	1.4	1.7	6.0	10.6
*o*-Xylene	63	1.6 (1.6)	0.5	0.7	0.8	1.1	6.2
Styrene	6	0.6 (0.6)	0.1	0.1	0.4	0.8	2.3
Toluene	17	4.7 (3.8)	0.5	1.3	4.3	6.0	16.5
1,2,4-Trimethylbenzene	3	0.5 (0.3)	0.1	0.3	0.5	0.6	1.1
Naphatalene	88	0.3 (0.1)	0.2	0.2	0.2	0.3	0.6
Phenol	3	2.3 (1.4)	0.2	1.2	2.1	3.1	5.9
**Aliphatic hydrocarbons**							
n-Hexane	88	1.9 (6.4)	0.6	0.7	0.8	0.9	39.0
Cyclohexane	85	0.9 (0.5)	0.6	0.6	0.8	0.9	2.6
n-Heptane	91	0.9 (0.9)	0.6	0.6	0.7	0.8	6.1
n-Decane	100	-	-	-	-	-	-
n-Undecane	97	3.8 (0.6)	2.9	3.3	3.8	4.1	5.5
**Halogenated hydrocarbons**							
1,1,1-Trichloroethane	34	0.6 (1.2)	0.1	0.2	0.3	0.6	6.7
Trichloroethylene	71	0.3 (0.3)	0.1	0.2	0.2	0.3	1.7
Chloroform	6	6.3 (5.3)	0.2	1.9	5.6	10.4	23.8
1,4-Dichlorobenzene	40	0.2 (0.2)	0.1	0.1	0.1	0.3	1.1
1-Bromopropane	100	-	-	-	-	-	-
**Alcohols**							
Ethanol	6	928 (958)	0.3	327	495	1297	3956
Isopropanol	14	47.9 (52.2)	0.7	4.5	20.3	87.8	174
Propan-1-ol	3	5.9 (5.6)	0. 5	2.9	4.1	5.5	24.9
2-Ethyl-1-hexanol	11	3.1 (2.3)	0.1	1.6	2.2	4.9	8.8
**Aldehydes**							
Acrolein	14	4.7 (4.4)	0.1	1.3	3.9	7.0	18.1
Formaldehyde	17	5.8 (4.0)	1.5	2.2	5.1	8.7	14.8
Acetaldehyde	20	5.7 (4.3)	1.0	2.6	4.1	9.1	16.2
Propionaldehyde	100	-	-	-	-	-	-
Butyraldehyde	100	-	-	-	-	-	-
Isovaleraldehyde	74	2.2 (1.6)	1.0	1.3	1.5	1.8	5.9
Valeraldehyde	100	-	-	-	-	-	-
Hexaldehyde	68	1.9 (0.9)	1.0	1.3	1.5	2.6	4.2
*o*-Tolualdehyde	100	-	-	-	-	-	-
*m*-Tolualdehyde	100	-	-	-	-	-	-
*p*-Tolualdehyde	100	-	-	-	-	-	-
**Ketones**							
Acetone	11	22.6 (20.6)	0.1	5.9	18.5	32.6	82.3
2-butanone	37	8.7 (32.6)	0.1	0.5	0.7	1.6	174
Cyclohexanone	48	3.3 (5.4)	0.1	0.1	0.9	4.1	20.1
**Ethers**							
Ether	43	75.6 (157)	0.6	0.8	2.5	50.0	678
2-Ethoxyéthanol	94	0.8 (0.2)	0.6	0.6	0.8	0.8	1.6
2-Butoxyéthanol	100	-	-	-	-	-	-
2-Phenoxyethanol	8	1.4 (2.1)	0.1	0.3	0.5	1.8	11.6
**Terpenes**							
Limonene	66	8.7 (18.6)	2.9	3.7	4.2	6.6	113

The results showed that the contamination of indoor air was dominated by alcohols, with arithmetic means (± SD) ranged from 3.1±2.3 µg/m^3^ for 2-ethyl-1-hexanol to 928±958 µg/m^3^ for ethanol. The concentrations of ether (75.6±157 µg/m^3^) and acetone (22.6±20.6 µg/m^3^) were relatively high compared to other compounds. A few target compounds, including n-decane, 1-bromopropane, propinaldehyde, butyraldehyde, valeraldehyde, tolualdehyde (o,m,p) and 2-butoxyethanol were not detected.

For a large number of compounds, including some halogenated hydrocarbons, alcohols, ketones and ethers, the SD was greater than or equal to the arithmetic mean, indicating a large variability in concentrations.

**Figure 1:**
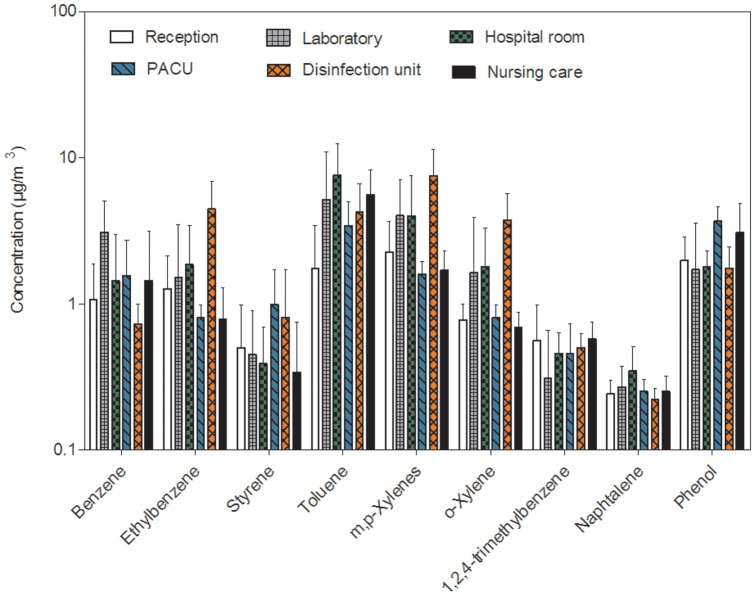
Comparison of aromatic hydrocarbons concentrations (arithmetic mean ± SD on a logarithmic scale) in the six sampling sites.

For aromatic hydrocarbons, Figure 1 presents the mean concentrations (± SD) of compounds measured in different sites.

The concentrations of aromatic hydrocarbons were lower than 10 µg/m^3^ (mean). A concentration gradient (toluene and m,p-xylenes>benzene and ethylbenzene>1,2,4-trimethylbenzene and naphthalene). The highest concentrations of benzene were measured in the parasitology laboratory. The highest concentrations of ethylbenzene and xylenes (o,m,p) were measured in the flexible endoscope disinfection unit. Concentrations of 1,24-trimethylbenzene, naphtalene and phenol measured in different sites were not significantly different (p>0.05).

**Figure 2:**
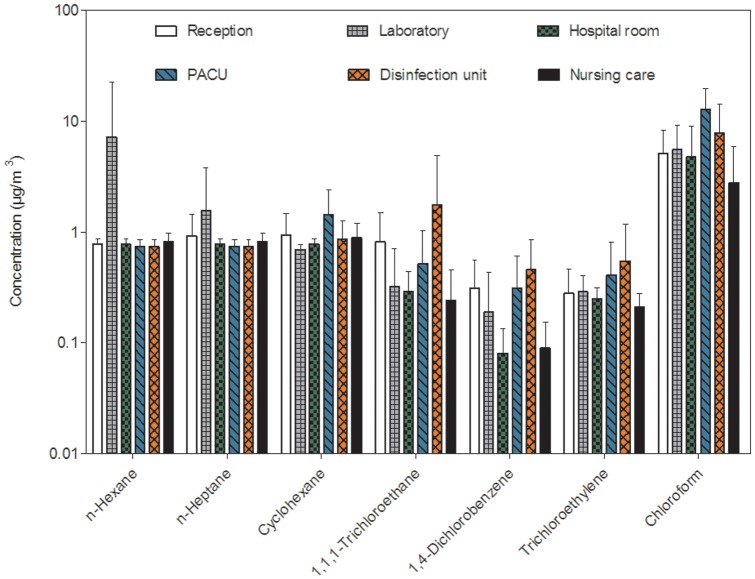
Comparison of aliphatic and halogenated hydrocarbons concentrations (mean ± SD on a logarithmic scale) in the six sampling sites.

Aliphatic and halogenated hydrocarbons were very few detected, as shown by the proportion of concentrations lower than the LOQ that ranged from 88 to 100% (Table 1). Figure 2 shows the mean concentrations (± SD) of these compounds measured in the different sites.

Concentrations of chloroform (close to 10 µg/m^3^) were significantly higher than those found for other aliphatic hydrocarbons, except in the parasitology laboratory. Concentrations of n-heptane and cyclohexane were on a similar ordered, with concentrations around 1 µg/m^3^. For 1,1,1-trichlorethane, 1,4-dichlorobenzene and trichloroethylene, the highest concentrations were found in the flexible endoscope disinfection unit, while the lowest were observed in the patient room and the nursing care. The highest concentrations of these compounds were found in sites where intensive disinfection of surfaces was performed.

**Figure 3:**
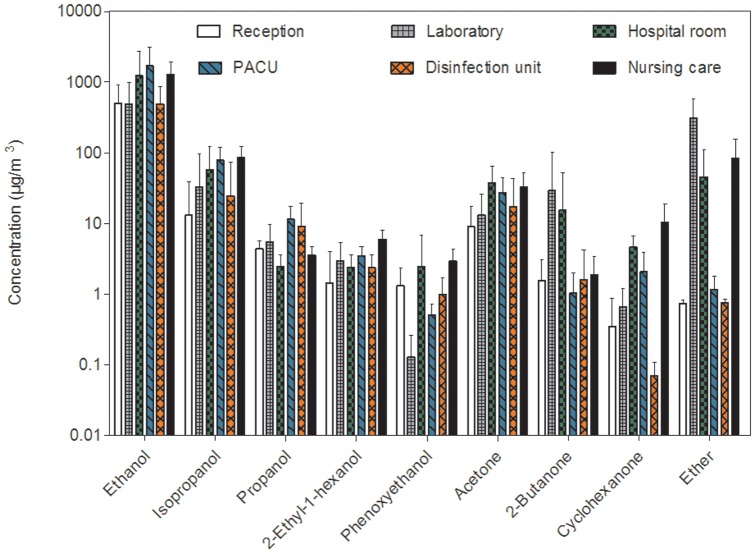
Comparison of alcohols, ketones and ethers concentrations (mean ± SD on a logarithmic scale) in the six sampling sites.

Figure 3 presents the mean concentrations (± SD) of alcohols, ketones and ethers measured in the different sites.

Concentrations of alcohols found in indoor air were close to 1000 µg/m^3^ for ethanol, 100 µg/m^3^ for isopropanol and less than 10 µg/m^3^ for other compounds. The highest concentrations of ethanol and isopropanol were measured in three sites (PACU, patient room and nursing care). For other alcohols, the highest concentrations were found in the nursing care. Concentrations of ketones found were close to 25 µg/m^3^ for acetone, 14 µg/m^3^ for 2-butanone and 5 µg/m^3^ for cyclohexanone. As for ethanol and isopropanol, concentrations of acetone were ubiquitous and on similar level in different sites. However, concentrations of 2-butanone and cyclohexanone were widely variable between different sites. Concentrations of 2-butanone observed in the patient room and the parasitology laboratory were higher than those found in other sites. The highest concentrations of cyclohexanone were found in the nursing care. Concentrations of ether were also widely variable between sampling sites. Concentrations found in the parasitology laboratory, the hospital room and the nursing room were higher than those measured in other sites.

**Figure 4:**
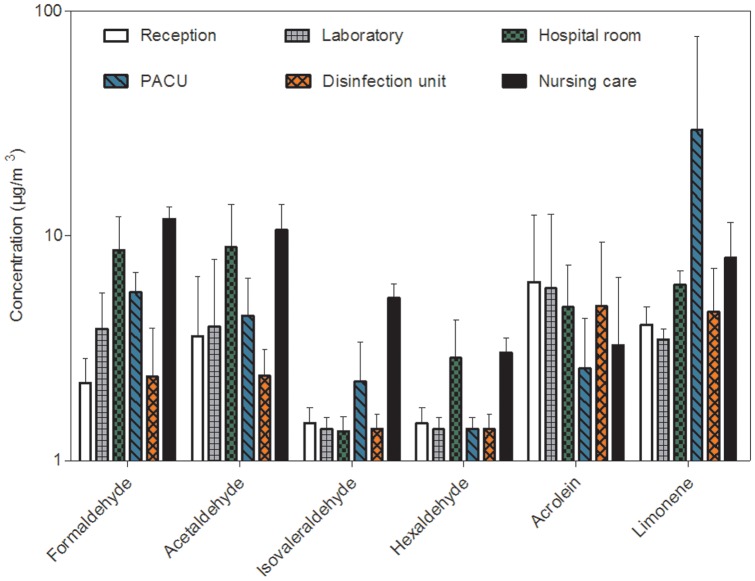
Comparison of aldehydes and terpenes concentrations (arithmetic mean ± SD on a logarithmic scale) in the six sampling sites.

For aldehydes, propionaldehyde, valeraldehyde and tolualdehydes (o,m,p) were not detected. Figure 4 shows the mean (± SD) concentrations of aldehydes and terpenes measured in different sites.

Our results show that concentrations of formaldehyde and acetaldehyde were equally distributed between the different sites. The highest concentrations were measured in the nursing care and the patient room. Concentrations of acrolein were slightly higher in the reception hall and the parasitology laboratory than in other sites. Among terpenes, the limonene was only measured and more than half of the concentrations (66%) were below LOQ. The mean concentrations of limonene found in indoor air were close to 6 µg/m^3^ with a low SD (around 1.3 µg/m^3^), except in the PACU where high concentrations were measured (30.5±46.7 µg/m^3^). In this site, limonene was quantified only from air samples collected during cleaning and disinfection of surfaces.

Overall, the nature of the contamination was not significantly different between sampling sites (p>0.05). The reception hall was initially selected as reference, because no health activities are performed in this area. Results showed that concentrations of VOCs were similar to other sites. In addition, there is no difference between VOCs concentrations measured in the morning and those measured in the afternoon (p>0.05).

### Qualitative evaluation of air samples

Complementary to the quantitative analysis, a qualitative analysis of other compounds, not selected as target compounds, was carried out in indoor air of sampling sites. Table 5 presents the compounds identified in at least one air sample in different sites.

**Table 5:** Other compounds identified (CAS number) in at least one air sample in the parasitology laboratory, the patient room, the nursing care, the post-anesthesia care unit (PACU), the flexible endoscope disinfection unit and the reception.

Compounds	CAS no.	Laboratory	Patient room	Nursing care	PACU	Disinfection unit	Reception
**Halogenated ethers**							
Desflurane	57041-67-5				×		
Sevoflurane	1000308-79-8		×	×			
**Aliphatic hydrocarbons**							
n-Butane	106-97-8		×	×		×	×
**Aldehydes**							
Benzaldehyde	100-52-7		×	×			
**Halogenated hydrocarbons**							
Chloroethane	75-00-3	×	×	×	×	×	×
**Alcohols**							
Benzylalcohol	100-51-6		×	×			
**Esters**							
Ethylacetate	141-78-6		×			×	
**Siloxanes**							
Hexamethyldisiloxane	107-46-0				×		
Octamethyltrisiloxane	107-51-7				×		
Decamethylcyclopentasiloxane	541-02-6				×		
**Terpenes**							
Camphor	76-22-2				×		
α-bisabolol	515-69-5				×		×
**Organic acids**							
Benzoic acid	65-85-0					×	

Results show that only a few numbers of compounds identified (13) were not quantitatively analyzed before. Most of these compounds were detected in the patient room, the nursing care and the PACU. Siloxanes were exclusively identified in the PACU. Anesthetic gases, sevoflurane and desflurane were found in the PACU as expected (Table 2). Sevoflurane was also found in the patient room. Ethylacetate were found in the patient room and the flexible endoscope disinfection unit. Butane was identified in all sites, except the parasitology laboratory and the PACU. Benzaldehyde and benzoic acid were observed in the patient room and the nursing care. Camphor was found only in the PACU. Alpha-bisabolol was identified in the reception and in the PACU.

## Discussion

### Possible sources and health effects of VOCs

First of all, for all target compounds, concentrations measured in indoor air were largely below the occupational exposure limit values set in France, European Union and United States of America. In addition, concentrations of benzene (1.5±1.5 µg/m^3^) and formaldehyde (5.8±4.0 µg/m^3^) were lower than the guideline values set in French public building (Décret n°2011-1727).

Concentrations of aromatic hydrocarbons measured are similar or lower than those found in residential and non-residential indoor environments [26]–[28]. Previous studies have reported that these compounds are mainly emitted from building materials and outside traffic [26], [29]. In addition, SD were less than or equal to arithmetic means, indicating a low variability in concentrations, except for styrene. Chronic exposure to benzene may induce genotoxic, immunological and hematological effects [30] and there is no safe level of exposure. However, based on WHO guidelines for indoor air quality [30], average concentration of benzene measured in our study (1.5±1.5 µg/m^3^) would be associated with an excess lifetime risk of 1/100 000. Exposure to naphthalene may induce respiratory tract lesions [30]. The guideline value of 10 µg/m^3^ recommended is far above concentrations of naphthalene observed (0.3±0.1 µg/m^3^).

Aliphatic hydrocarbons are contained in paints, adhesives and building materials [29]. Acute exposure to these compounds may affect the central nervous system and induce drowsiness and dizziness.

For halogenated hydrocarbons, the temporal variability in concentrations observed (SD higher or equal to arithmetic mean) is possibly induced by specific health activities carried out in sampling sites. The highest concentrations of these compounds were found in sites where intensive disinfection of surfaces was performed. Concentrations of trichloroethylene found were in accordance with concentrations of observed in homes [31], [32]. This compound is present in paints and adhesives. Concentrations measured in sampling sites (from 0.06 to 1.74 µg/m^3^) were below the concentration associated with an excess lifetime cancer risk of 1/1 000 000 (2.3 µg/m^3^) [30]. For chloroform, concentrations were below the 8 hours time weighted-average concentrations of 2.5 mg/m^3^, 10 mg/m^3^ and 49 mg/m^3^, established by Deutschland, European Union and US, respectively. 1,4-dichlorobenzene is used in cleaning products and air freshener. Exposure to high concentrations of 1,1,1-trichloroethane and 1-bromopropane may affect the central nervous system and induce irritations of the respiratory tract, but there is a lack of knowledge regarding human toxicity.

For alcohols, the concentration gradient (ethanol>isopropanol>propan-1-ol) observed in air samples is in accordance with the proportion of their use in the formulation of ABHRs [14]. Temporal variations (elevated SD) in concentrations are probably due to the planning of use of alcohol-based products. The highest concentrations of ethanol and isopropanol were measured in three sites (PACU, patient room and nursing care). This result is in accordance with the preliminary interviews [21]. Four alcohol-based products were listed in these rooms (Table 2). During hand rubbing, workers are exposed to alcohols through inhalation and dermal contact. Although the consumption of alcoholic beverages is known to induce adverse health effects, studies conducted dermal and pulmonary absorption of alcohols reported that blood ethanol concentrations were below those known to be harmful in humans [33], [34]. However, few studies have found that intensive use of ethanol-based hand rubs and mouthwash leads to false-positive results related to ethanol consumption [35], [36]. In addition, exposure of workers to a sudden change in ethanol concentrations from 0 to 3.6 mg/L may cause temporary irritation [37]. A particular attention should be paid to pregnant healthcare workers or patients, for whom exposure to alcohol may put the baby at risk of developing fetal alcohol spectrum disorders.

Concentrations of acetone measured are higher than those found in public building and houses [26]. There is a lack of data regarding human toxicity of acetone, 2-butanone and cyclohexanone.

Aldehydes are mainly emitted from building and decoration materials [29]. Differences in building age of different sites could explain the large spatial variability in concentrations measured. In addition, concentrations found are lower than those reported in other indoor environments [26]. Among aldehydes, adverse health effects of formaldehyde are well known. Exposure to this compound may increase the risk to develop a myeloid leukemia [38]. Levels of formaldehyde found in the six sampling sites were lower than the short-term guideline value of 100 µg/m^3^ (30-minutes average concentration), and the long-term guideline value of 200 µg/m^3^ established by WHO [30].

Exposure to limonene, commonly used as a fragrant in cleaning products, may induce irritative and allergenic effects.

Qualitative analysis of air samples led to the identification of other compounds, not selected as target compounds in sampling sites. Siloxanes are used as ingredients in the formulation of personal care products (for skin and hair) and pharmaceutical products (liquid and gastric bandages) [39]. There is no data available on the toxicity of this class of chemicals in humans. Sevoflurane found in the patient room, is possibly emitted from exhaled air of patients after post-anesthesia care. There is a lack of knowledge regarding the occupational exposure to halogenated ethers. Ethylacetate found in the patient room and the flexible endoscope disinfection unit could be emitted from cleaning products where it is used as perfuming agent. It could also be formed by reaction between ethanol and acetic acid used in the disinfection unit. Exposure to this compound may affect the central nervous system. Camphor is an odorous terpens used in many cleaning and medical products due to its anti-microbial and local anesthetic properties [40], [41]. Alpha-bisabolol is an ingredient of skin protection creams due to its anti-irritant, anti-inflammatory and anti-microbial properties [42]. There is no data available on the toxicity of butane, benzaldehyde, benzoic acid and α-bisabolol in humans

Multiple sources specific or not to health activities conducted in the teaching hospital induce a complex mixture of VOCs. Finally, there is no evidence for considering that exposure of VOCs, at concentrations measured, poses a health threat for healthcare workers and patients. However, special attention should be paid to possible health effects induced by exposures to such a mixture of VOCs.

### Strengths and limitations

So far as we are aware, this study is the first assessment of the nature of the chemical contamination in hospitals, with the measurement of a large panel of VOCs (42). Our study was designed to measure VOCs in indoor air at different locations, where various health activities are conducted. Previous studies addressing the issue of healthcare workers exposure were focused on a few activities at risk such as disinfection of medical device with glutaraldehyde or formaldehyde [5], [6], solvent handling in pathology laboratory [8], [39] or use of anesthetic gases in operating rooms [7]. In addition, air samples were collected in the morning and in the afternoon during 3 days, in order to have a first idea on the temporal variability in VOCs concentrations, and on the exposure of healthcare workers and patients.

The main limitation of our study arises from the fact that only one hospital was investigated. VOCs identified, concentrations levels found and possible sources of contamination are specific to the teaching hospital studied. However, as chemical products such as cleaning/disinfecting products or alcohol-based products are the primary sources of contamination in hospitals, concentrations levels of certain VOCs would be on a similar order in other hospitals. But, VOCs are also released from other sources, such as building materials or outside traffic, which are not related to health activities and specific to the hospital building and its surronding environment. In order to differentiate compounds originating from outdoor and those emitted indoor, determination of outdoor air VOC concentrations is required. Here, outdoor air samples were not collected, because the primary purpose of our study is to evaluate the nature of the indoor contamination. Other limitations are related to the sampling method used. Measurements of VOCs concentrations were carried out from air samples collected at fixed points. Results found represent ambient concentration of VOCs and does not reflect the real exposure of workers and patients. Stationary sampling does not take into account the duration of exposure and the travels of people between the rooms. However, this sampling method was chosen, in a view of the identification of VOCs dynamic. Personal sampling does not provide data on peak exposure because air samples are usually collected during a work shift (8 hours).

### Comparison of VOCs concentrations measured in previous studies

Table 6 summarizes concentrations of VOCs found in our study and those previously reported in different works in hospitals. Even if we have measured or detected more VOCs in six sampling locations than in previous studies, glutaraldehyde frequently cited was not found in our work because no more used. In some other sites such as operating rooms investigated in other studies indoor air was contaminated mainly by anesthetic gases and by aldehydes, oxides and alcohols [7], [8]. Formaldehyde and glutaraldehyde used as disinfectants of medical devices were also observed, concentrations of formaldehyde being very close in both studies. Most of the previous works have measured VOCs concentrations in disinfection rooms where medical devices are disinfecting and sterilizing with glutaraldehyde or formaldehyde. Concentrations of glutaraldehyde were widely variable between studies [6], [5], [43], [44], The difference being probably due to the type of ventilation. The highest concentrations were observed in rooms with natural ventilation [5]. Aspiration hood or general ventilation decreased significantly concentrations levels [44]. Concentrations of formaldehyde measured in our study are in accordance with those reported from the study of Lü et al. [12]. However, concentrations reported from the study of Ghasemkhani et al. [8] were 10 times higher. In our study, the disinfection room is equipped with a general ventilation system delivering a high air flowrate (1,170 m^3^/h). Glumbakaite et al. [5] reported much higher concentrations of alcohols than those measured in our study. The authors measured concentrations of alcohols before, during and after disinfection with alcohols, while in our study alcohol-based products were not used for disinfection of medical devices. In addition, concentrations levels of these compounds were probably greatly influenced by the ventilation rate. Concentrations of aromatic hydrocarbons and other aldehydes observed in our study were also lower than those reported by Lü et al. [12]. Lü et al. [12] have reported that indoor/outdoor concentrations ratio of aromatic hydrocarbons and aldehydes were higher than 1, indicating indoor sources for these compounds. Differences among concentrations of aldehydes may be due to the lower number of compounds detected (10) in our study than in the study of Lü et al. [12]. In addition, ozone being also used as disinfectant, some aldehydes might have been formed by the reaction between ozone and carbonyls [12]. In laboratories, publications have reported very high concentrations of formaldehyde [8], [43]. Both studies were performed in pathology laboratories where formaldehyde is commonly used and reported that the contamination might be due to the lack of adequate ventilation in the rooms studied (no local exhaust ventilation). In our study, the parasitology laboratory is equipped with a biosafety cabinet, preventing the emission of pollutants into the room. Concentrations of aromatic hydrocarbons measured in the nursing room, were lower in our study than those reported by Kang et al. [45]. In this study, sampling devices were attached on the nurses' suite collar, collecting air samples from the breathing zone. Concentrations measured were probably affected by indoor air contamination of other rooms frequented by the nurses traveling during the sampling period (up to 72 hours).

**Table 6:** Comparison of VOCs concentrations measured with those reported to selected references.

Sampling site	Chemical family (number of compounds)	Concentration^a^ (µg/m^3^)	City (Country)	Reference
Operating rooms	Anesthetic gases (2)	2,362 [ND^b^ – 9,652]	Athens (Greece)	[7]
	Aromatic hydrocarbons (5)	239 [21–564]	Athens (Greece)	[7]
	Formaldehyde	288 [ND^b^ – 1,040]	Athens (Greece)	[7]
		310 [12–1,030]	Tehran (Iran)	[8]
	Glutaraldehyde	207 [ND^b^ – 458]	Athens (Greece)	[7]
	Other aldehydes, oxides, alcohols	1,920 [107–5,268]	Athens (Greece)	[7]
	Other	3,846 [31–41,255]	Athens (Greece)	[7]
Disinfection rooms	Glutaraldehyde	208 [60–840]	Chieti (Italy)	[6]
		2220 [340–6,910]	Vilnius (Lithuania)	[5]
		1,430 [410–3,270]	Osaka (Japan)	[39]
		296.2±246.0	Firenze (Italy)	[40]
	Formaldehyde	1.80±0.7	Guangzhou (China)	[12]
		160 [12–810]	Tehran (Iran)	[8]
		2.6±1.2	Rennes (France)	Our study
	Other aldehydes (18)	36.7±11.6	Guangzhou (China)	[12]
	Other aldehydes (10)	18.3±3.6	Rennes (France)	Our study
	Alcohols (2)	368,150 [2,400–469,200]	Vilnius (Lithuania)	[5]
	Alcohols (5)	519.2±405.6	Rennes (France)	Our study
	Aromatic hydrocarbons (5)	357.5±85.6	Guangzhou (China)	[12]
	Aromatic hydrocarbons (10)	24.1±11.7	Rennes (France)	Our study
	Aliphatic hydrocarbons (5)	13.6±2.4	Rennes (France)	Our study
	Halogenated hydrocarbons (5)	11.4±9.9	Rennes (France)	Our study
	Ketones (3)	19.1±26.2	Rennes (France)	Our study
	Ethers (3)	7.3±1.3	Rennes (France)	Our study
	Limonène	5.9±1.9	Rennes (France)	Our study
Laboratories	Formaldehyde	1,180 [40–4,910]	Tehran (Iran)	[8]
		2,820 [980–6,130]	Osaka (Japan)	[39]
		4.0±1.5	Rennes (France)	Our study
	Other aldehydes (10)	19.7±5.7	Rennes (France)	Our study
	Alcohols (5)	670.8±727.6	Rennes (France)	Our study
	Aromatic hydrocarbons (10)	20.9±16.2	Rennes (France)	Our study
	Aliphatic hydrocarbons (5)	20.0±18.8	Rennes (France)	Our study
	Halogenated hydrocarbons (5)	8.5±5.0	Rennes (France)	Our study
	Ketones (3)	45.6±83.6	Rennes (France)	Our study
	Ethers (3)	316.6±270.0	Rennes (France)	Our study
	Limonène	4.9±0.6	Rennes (France)	Our study
Nursing rooms	Aromatic hydrocarbons(2)	42.1±10.4	Buchean (South Korea)	[41]
	Aromatic hydrocarbons(10)	14.8±7.9	Rennes (France)	Our study
	Alcohols (5)	1384.8±702.3	Rennes (France)	Our study
	Aliphatic hydrocarbons (5)	14.6±1.4	Rennes (France)	Our study
	Halogenated hydrocarbons (5)	4.4±3.7	Rennes (France)	Our study
	Formaldehyde	11.9±1.6	Rennes (France)	Our study
	Other aldehydes (10)	31.4±6.1	Rennes (France)	Our study
	Ketones (3)	44.8±29.2	Rennes (France)	Our study
	Ethers (3)	89.3±73.9	Rennes (France)	Our study
	Limonène	8.6±2.8	Rennes (France)	Our study
Pharmacy rooms	Formladehyde	4.1±1.6	Guangzhou (China)	[12]
	Other aldehydes (18)	39.6±5.9	Guangzhou (China)	[12]
	Aromatic hydrocarbons (5)	552.5±376.9	Guangzhou (China)	[12]

a Concentrations are expressed as arithmetic mean ± SD or arithmetic mean [min – max] depending of data available.

b Not detected.

### Future research

Exposure of healthcare workers and patients to chemical products depend on product formulations, product application procedures, location where products are used, and on the use of protection devices in some cases. Future studies need to explore exposure to healthcare workers and patients through measurements of exposure concentrations by personal sampling or by coupling ambient concentrations with time-activity data. In order to assess a whole organic contamination of indoor air in hospital, exposure to SVOCs, such as phtalates, released from PVC-based medical devices or building materials must also be considered. Finally, our results have to be confirmed in a multicentric manner and research efforts must be planned with regard to the possible health effects induced after inhalation exposure to a complex mixture of chemical compounds.

## Conclusions

This study is a first integrated approach of the assessment of the nature of COVs contamination in hospitals, consisting in measuring simultaneously more than 40 chemicals compounds in six sampling locations. The main VOCs measured were alcohols (ethanol, isopropanol), ethers (ether), ketones (acetone), terpenes (limonene), and halogenated hydrocarbons (chloroform). Concentrations of VOCs were very variable between sampling sites. For certain compounds (alcohols, ethers, terpenes, ketones) a significant temporal variability in concentrations levels was also observed. These variations are mainly due to multiples sources of emission. Although concentrations of all compounds measured were largely below occupational exposure limits, healthcare workers and patients may be exposed to a complex mixture of VOCs. In hospitals, the use of chemical products is the primary source of contamination as a high number of products, including cleaning and disinfectants products, alcohol-based products, pharmaceutical products and antiseptics, anesthetic gases, and laboratory products are used for different activities.
